# Top ten tips in managing ANCA vasculitis

**DOI:** 10.1093/ckj/sfae389

**Published:** 2024-11-30

**Authors:** Min Hui Tan, David Jayne

**Affiliations:** Department of Medicine, University of Cambridge, Cambridge, United Kingdom; Department of Nephrology, Hospital Kuala Lumpur, Kuala Lumpur, Malaysia; Department of Medicine, University of Cambridge, Cambridge, United Kingdom

**Keywords:** ANCA vasculitis, avacopan, cyclophosphamide, rituximab

## Abstract

Diagnosing and managing antineutrophil cytoplasmic antibody (ANCA)-associated vasculitis (AAV) remain a challenge for many clinicians, due to the complexity of the disease manifestations and its treatment. There has been a paradigm shift in ANCA vasculitis management, where treatment incorporates both emergency life- and organ-saving procedures and longer-term care to manage relapse and co-morbidity risk and the complications of organ damage. Here, we highlight 10 key tips for the management of ANCA-associated vasculitis based on current evidence and clinical experience. First, we advise making the diagnosis as early as possible, emphasizing the importance of using high-quality ANCA assays. Second, we recommend the use of glucocorticoids in combination with rituximab and/or cyclophosphamide as induction therapy. Third, plasma exchange should be considered in patients with severe renal impairment and diffuse alveolar haemorrhage. We advise the use of rapidly reducing glucocorticoid regimens and advocate consideration of avacopan early in the disease course. We recommend the use of rituximab as maintenance therapy and routine monitoring of kidney function, proteinuria, ANCA and immunoglobulin levels at baseline and during follow-up. The use of prophylactic antibiotics in susceptible patients and timely vaccination schedules is discussed. Rituximab is the preferred immune suppressive for treatment of relapse. Finally, we recommend switching treatment modalities in patients whose vasculitis is refractory to induction therapy and to consider plasma exchange in selected patients. These key tips aim to provide the necessary guidance to improve patient outcomes and reduce adverse events.

## TIP 1. MAKE THE DIAGNOSIS AS EARLY AS POSSIBLE

Diagnosing antineutrophil cytoplasmic antibody (ANCA) vasculitis is challenging due to its varied clinical manifestations and overlapping features with other autoimmune diseases. Classification of ANCA vasculitis has been facilitated by the development of the American College of Rheumatology/European Alliance of Associations for Rheumatology (ACR/EULAR) classification criteria for granulomatosis with polyangiitis (GPA), microscopic polyangiitis (MPA), and eosinophilic granulomatosis with polyangiitis (EGPA) [1–3]. While these criteria aid in disease recognition and classification, they are not diagnostic criteria and their performance in the diagnosis of ANCA vasculitis has not been assessed. Detailed medical history and physical examination and exclusion of other causes for the clinical presentation remain essential steps in clinical assessment [4, 5].

It is not uncommon for patients to present with hypoxic pulmonary haemorrhage and rapidly progressive glomerulonephritis, typically termed ‘pulmonary renal syndrome’. They rarely have nephrotic range proteinuria but the level of hypoalbuminaemia together with raised C-reactive protein may reflect the severity of the ongoing active inflammatory process [6]. Age at presentation has associations with disease characteristics and may influence treatment decisions [7].

The use of high-quality antigen-specific assays for proteinase-3 (PR3)-ANCA and myeloperoxidase (MPO)-ANCA as the primary screening method has been advocated, but a positive result is not diagnostic by itself. Similarly, a negative result cannot exclude the diagnosis of ANCA vasculitis. The use of indirect immunofluorescence is advised if suspicion remains high despite negative results, but this test alone has relatively low specificity for the diagnosis of ANCA-associated vasculitis (AAV) [8, 9].

The presence of PR3 or MPO-ANCA is highly predictive of pauci-immune glomerulonephritis in an appropriate clinical presentation, warranting early treatment for patients who present with organ- or life-threatening disease [10]. The hallmark of histological findings in renal vasculitis is a pauci-immune, focal necrotizing and crescentic glomerulonephritis. However, the diagnosis of ANCA vasculitis should not be delayed for biopsy as treatment delays worsen prognosis and renal outcomes, and, infrequently, a more chronic kidney course is seen with fibrosis the predominant feature on biopsy.

While renal biopsy is definitive, it is not essential in typical presentations with positive MPO- or PR3-ANCA with low suspicion for secondary vasculitis or mimics [11]. On the contrary, the role of biopsy in guiding prognostication and aiding prediction of renal recovery cannot be dismissed. A small study of renal biopsies of 29 patients highlighted the importance of confirmation of disease activity using histological samples compared with proteinuria and haematuria. Incorporation of biopsy findings and glomerular filtration rate (GFR) in the ANCA Renal Risk Score (ARRS) has also been validated to help discriminate the risk of end-stage kidney disease (ESKD). A revision of the ARRS, the ANCA Kidney Risk Score (AKRiS), combines kidney function with histopathological findings to predict kidney survival probability [12–15].

## TIP 2. USE GLUCOCORTICOIDS IN COMBINATION WITH RITUXIMAB AND/OR CYCLOPHOSPHAMIDE FOR INDUCTION TREATMENT OF REMISSION

The use of glucocorticoids is part of the standard of care for induction of remission. A typical dosing regimen includes three pulses of intravenous (IV) methylprednisolone (total dose of 1000–3000 mg) followed by rapid tapering of oral prednisolone [16]. Reduced dose regimens have gained favour in recent years owing to fewer infectious complications while maintaining good efficacy (see Tip 4).

Rituximab is increasingly preferred for all GPA and MPA patients, including those with moderate to severe kidney impairment, and has been recommended in various guidelines [9, 17, 18]. It is a chimeric, monoclonal antibody targeting CD20 cells, originally developed for non-Hodgkin's lymphoma and also approved for rheumatoid arthritis. Currently there are two recommended dosing regimens, the first being 375 mg/m^2^ weekly for 4 weeks and the second being two fixed doses of 1 g given 2 weeks apart. A meta-analysis published in 2021 revealed no difference in terms of efficacy or safety between the two rituximab regimens [19]. The second regimen has been preferred for its ease and convenience of administration for patients. Prescribers need to be aware of dosing adjustments for patients with nephrotic range proteinuria as they lose rituximab into the urine. Although rituximab is generally well tolerated, cases of progressive multifocal leucoencephalopathy and hepatitis B reactivation have been reported [20]. The RAVE trial demonstrated that its efficacy and safety for induction of disease remission, comparable to that of cyclophosphamide, and a further *post hoc* analysis showed similar outcomes for patients with renal involvement [21, 22]. This trend was also observed in the RITUXVAS trial, which had a longer follow-up duration of up to 24 months after rituximab withdrawal [23]. In another French trial emulation study, patients who received rituximab more frequently achieved remission compared with cyclophosphamide-treated patients [24].

On the other hand, cyclophosphamide remains the treatment of choice for patients who are unable to have rituximab. Being an alkylating agent, it can be administered orally or intravenously. The IV route uses a recommended dosing of 15 mg/kg at specific intervals of weeks 0, 2, 4, 7, 10, and 13 (and 16, 19, 21, and 24 if required) [16]. See Table 1 for dosing recommendations. Oral cyclophosphamide remains an option but is associated with a higher exposure to cyclophosphamide and risk of urothelial and other malignancies. Prescribers need to be aware of dose adjustments with both oral and IV regimens for patients with renal impairment and dose-dependent complications of malignancies, bone marrow failure, myelodysplasia, and premature ovarian failure; thus thorough counselling with the patient is mandatory. While both oral and IV regimens were effective in the CYCLOPS trial, the IV route utilizes a lower cumulative dose, facilitates bladder protection by pre-hydration, and has a lower risk of leucopenia. Over long-term follow-up, however, the relapse rate is higher with IV cyclophosphamide, reflecting reduced cumulative exposure [25, 26].

**Table 1:** Cyclophosphamide regimen.

Route	Dose	Special considerations
Intravenous	15 mg/kg at weeks 0, 2, 4, 7, 10, 13 (16, 19, 21, 24 if required)	If eGFR <30 mL/min/1.73 m^2^ reduce by 2.5 mg/kg If age 60–69 years use 12.5 mg/kg If age >70 years use 10 mg/kg
Oral	2 mg/kg/day for 3–6 months	If eGFR <30 mL/min/1.73 m^2^ reduce by 0.5 mg/kg/day If age 60–69 years use 1.5 mg/kg/day If age >70 years use 1 mg/kg/day

Adapted from [16]

The combination therapy of cyclophosphamide and rituximab is gaining interest as this regimen reduces glucocorticoid exposure and minimizes cyclophosphamide toxicity. Single-centre studies from the UK and the Netherlands have demonstrated safety and efficacy of this regimen, albeit with different doses of cyclophosphamide, with an ongoing randomized controlled trial in the Netherlands investigating this further [27–29]. A regimen that has been developed from the RITUXVAS trial uses two doses of rituximab (1 g 2 weeks apart) and two doses of IV cyclophosphamide (15 mg/kg 2 weeks apart) [23].

## TIP 3. CONSIDER PLASMA EXCHANGE IN PATIENTS WITH SEVERE RENAL IMPAIRMENT AND DIFFUSE ALVEOLAR HAEMORRHAGE

Plasma exchange is an extracorporeal procedure that removes plasma from the patient after separating it from other blood constituents. It can remove ANCA and pro-inflammatory molecules from the blood and has been used as an adjunct to the conventional immunosuppressive therapy [30, 31].

The MEPEX trial, conducted in patients with severe renal impairment, found that plasma exchange improved renal recovery at 1 year [32]. However, the PEXIVAS trial showed no reduction in the incidence of the composite of death and ESKD over a longer disease course of an average of 3 years [33]. A meta-analysis concluded that plasma exchange, together with immunosuppressive therapies, reduces the risk of ESKD at 1 year but not mortality [34].

Both the current Kidney Disease—Improving Global Outcomes (KDIGO) and European Alliance of Associations for Rheumatology (EULAR) guidelines have considered plasma exchange for patients with serum creatinine >300 μmol/L, but they differ in its use for patients with diffuse alveolar haemorrhage [9, 16]. A *post hoc* analysis of patients with lung haemorrhage recruited to PEXIVAS found a strong trend to improved survival when plasma exchange was used in those presenting with hypoxia [35].

Access to and availability of plasma exchange services vary globally and this needs to be considered when deciding such a therapy regimen [36]. Selection of patients suitable for this therapy must be individualized, as they are often the sickest with guarded prognosis. Shared decision-making is essential as patients’ values and preferences may differ [37].

## TIP 4. USE A LOW-DOSE GLUCOCORTICOID REGIMEN

The LoVAS trial conducted in Japan has reported good long-term outcomes for a lower-dose glucocorticoid regimen starting at 0.5 mg/kg/day of prednisolone in a predominantly MPO-ANCA-positive population without severe kidney disease [38]. This was later confirmed by one of the largest trials in ANCA vasculitis, the PEXIVAS trial, which demonstrated non-inferiority of a more rapidly reducing dose regimen for the primary outcomes of ESKD or death, which had a reduced frequency of serious infections [33].

Further retrospective studies have reported good outcomes with a lower-dose glucocorticoid regimen [39], and a recent meta-analysis has demonstrated a better infection risk profile and similar efficacy of a low-dose glucocorticoid regimen, compared with the high-dose regimen, in inducing remission [40].

For more severe presentations, IV methylprednisolone (total dose of 1000–3000 mg) is recommended in the absence of firm evidence followed by rapid tapering of prednisolone as per the PEXIVAS trial (Table 2).

**Table 2:** Reduced-dose glucocorticoid regimen (based on PEXIVAS trial).

	Reduced dose regimen		
Weeks	<50 kg	50–75 kg	>75 kg
1	50	60	75
2	25	30	40
3–4	20	25	30
5–6	15	20	25
7–8	12.5	15	20
9–10	10	12.5	15
11–12	7.5	10	12.5
13–14	6	7.5	10
15–16	5	5	7.5
17–18	5	5	7.5
19–20	5	5	5
21–22	5	5	5
23–52	5	5	5

Adapted from [16].

## TIP 5. USE AVACOPAN EARLY

Avacopan is a newly approved oral complement 5a receptor inhibitor that prevents C5a-mediated neutrophil activation. It is dosed at 30 mg twice daily and can be given in combination with rituximab or cyclophosphamide for induction therapy, as part of a strategy to reduce glucocorticoid exposure [41, 42].

The ADVOCATE trial showed that avacopan was non-inferior to a tapered prednisone regimen at week 26 but superior to the prednisone taper in sustaining remission at week 52 [43]. It also showed that avacopan was associated with improved renal recovery, faster falls in albuminuria, less glucocorticoid toxicity, and better recovery of quality of life [44]. Emerging real-world evidence indicates that avacopan benefits patients with severe renal impairment (baseline eGFR <15 mL/min/1.73 m^2^), a cohort that was excluded from the ADVOCATE trial [45]. A limited multicentre case series reported favourable outcomes of using avacopan in patients with hypoxic pulmonary haemorrhage [46].

When avacopan is used as a component of induction therapy, it is recommended to follow the prednisone regimen of the ADVOCATE trial, where pre-existing prednisone was tapered to zero by 4 weeks.

## TIP 6. USE RITUXIMAB FOR THE MAINTENANCE TREATMENT OF REMISSION

Most patients achieve disease control within 3–6 months of conventional induction therapy, but a majority who achieve remission will relapse. The risk of relapse is higher in patients with PR3-ANCA positivity and in those who receive non-cyclophosphamide-based induction regimens [47, 48].

Rituximab is recommended for the maintenance of remission, with doses of 500–1000 mg every 6 months following two randomized controlled trials, MAINRITSAN and RITAZAREM. The MAINRITSAN trial studied new-onset patients induced with cyclophosphamide and dosed with rituximab at 500 mg every 6 months, while RITAZAREM focused on relapsing patients re-induced with rituximab and dosed at 1000 mg every 4 months. Both demonstrated clear superiority of rituximab over azathioprine regimens [9, 16, 18, 49]. Alberici *et al*. demonstrated the safety and efficacy of a fixed-interval rituximab maintenance regimen in preventing relapses [50]. Further analysis of the MAINRITSAN and RITAZAREM cohorts reported higher relapse rates after rituximab withdrawal [51]. There remains controversy over the role of ANCA and peripheral B-cell counts in determining re-dosing of rituximab or simply waiting for relapse and re-treating with rituximab when it occurs.

Other immunosuppressive medications, such as azathioprine, mycophenolate mofetil, and methotrexate, are alternatives to rituximab that reduce relapse risk. The REMAIN trial demonstrated increased relapse risk following azathioprine withdrawal at 18–24 months, and it is now recommended to continue for 2–4 years [52]. The additive value of low-dose glucocorticoids in reducing relapse risk has not been formally tested but is strongly suggested in systematic reviews of clinical trials [53]. The optimal duration of treatment in the remission phase is unknown, but the benefit of reduced relapse risk needs to be balanced against an increased risk of infection or malignancy.

**Figure 1:**
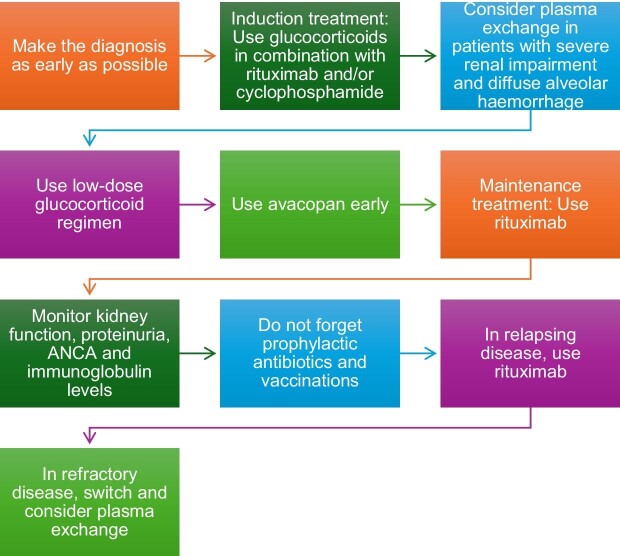
Top ten tips in managing ANCA vasculitis.

Treatment in the maintenance phase for some patients can be discontinued if they are already on chronic dialysis [16], as current literature suggests that these patients have a lower risk of relapse [54]. However, extrarenal relapses can still occur, so the decision on maintenance therapy should be individualized, considering factors such as disease duration, history of disease flares, ANCA status, presence of extrarenal or systemic manifestations, and risk of infections [55, 56].

## TIP 7. MONITOR KIDNEY FUNCTION, PROTEINURIA, AND ANCA AND IMMUNOGLOBULIN LEVELS

It is recommended at baseline and routine follow-up to monitor kidney function, proteinuria and ANCA levels during treatment and follow-up visits. For patients receiving rituximab, immunoglobulin levels should also be monitored. For patients with chronic kidney disease, we recommend referring to the latest KDIGO guidelines with regard to the roles of other reno-protective medications, such as renin–angiotensin system inhibitors (RASis), sodium-glucose cotransporter-2 inhibitors (SGLT2is), and mineralocorticoid receptor antagonists (MRAs) [57].

The evidence for the impact of kidney function on relapse risk remains heterogeneous but when combined with other parameters in a scoring system it has higher prognostic value [58–60].

A *post hoc* analysis of 571 patients from five European randomized clinical trials revealed the importance of monitoring for proteinuria and haematuria. Persistent proteinuria after induction therapy is associated with death and kidney failure while persistent haematuria independently predicts relapse [61].

The value of monitoring ANCA levels is highlighted by McClure *et al*., who observed that patients who achieved and maintained PR3-ANCA negativity after rituximab treatment had longer-lasting remission [62]. Persistent ANCA positivity at the time of disease remission as well as reappearance of ANCA level following clinical remission are linked to increased risk of relapse [63, 64]. Similar results were seen in a retrospective review of patients with MPO-ANCA vasculitis from the Mayo Clinic, whereby the risk of relapse was low even without maintenance therapy in patients with persistent MPO-ANCA negativity [65]. A meta-analysis of 20 studies identified that rising ANCA levels preceded a clinical relapse by 6–12 months [66].

Hypogammaglobulinaemia is common in patients treated with rituximab, with lower immunoglobulin G levels directly related to serious infections [67]. Monitoring of immunoglobulin levels for at least a year after stopping rituximab has been recommended [68]. The EULAR guidelines advised measuring immunoglobulin levels before each rituximab course [9]. Immunoglobulin replacement in patients with recurrent infections has been shown to reduce the rate of infections [69]. Indications for this therapy should be guided by the degree of hypogammaglobulinaemia, the presence of serious or recurrent infections, impaired antibody responses to polysaccharide antigens, and poor response to antimicrobial prophylaxis [68].

## TIP 8. DO NOT FORGET PROPHYLACTIC ANTIBIOTICS AND VACCINATIONS

Patients with ANCA vasculitis have increased susceptibility to infections and the risk factors include the type of induction treatment, cumulative exposure to glucocorticoids and cyclophosphamide, age, comorbidities, pulmonary involvement, and renal impairment. Most of the severe infections occur within 6 months of diagnosis, commonly affecting the upper respiratory tract [70–72].

**Figure 2:**
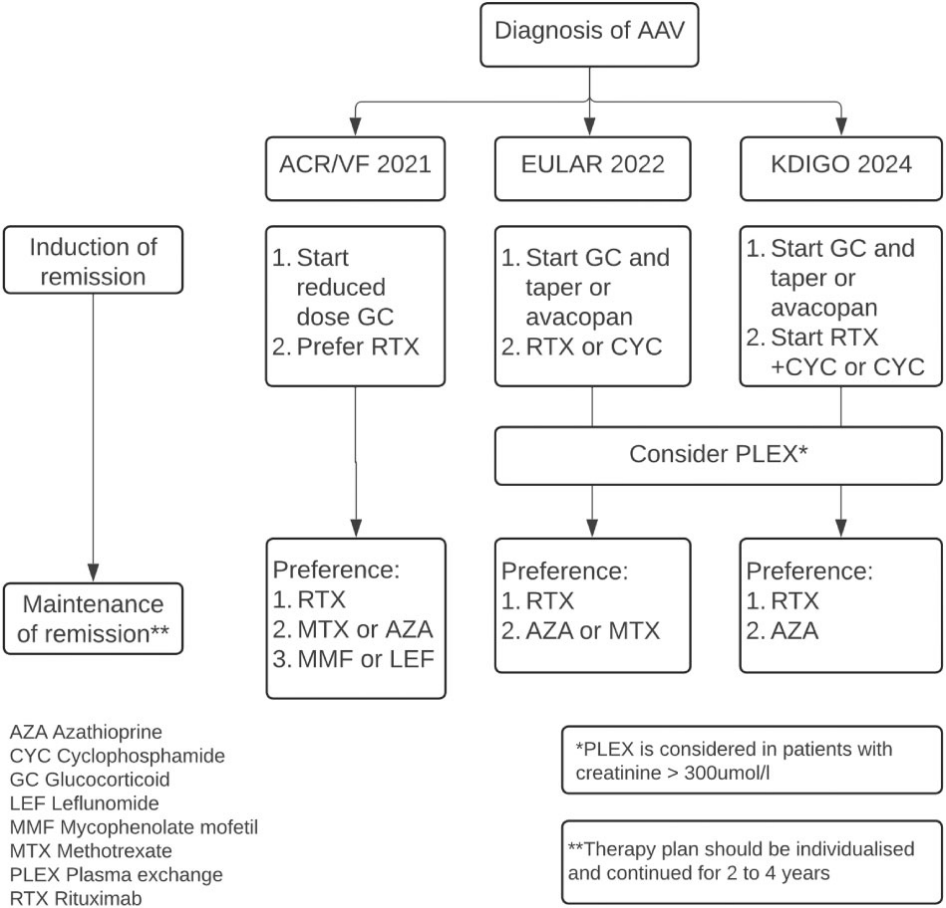
Management of ANCA vasculitis based on different guidelines. Adapted from [9, 16, 18, 52].

**Figure 3:**
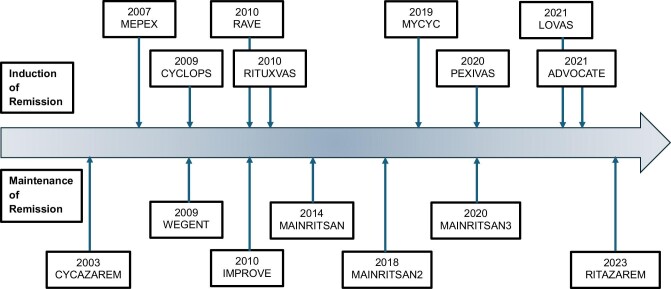
Landmark trials in ANCA vasculitis.

Analysis of the RAVE trial found a reduced rate of severe infections in patients who received co-trimoxazole prophylaxis and this has also been reported in observational studies [73, 74]. Hence, the guidelines have recommended prophylaxis against *Pneumocystis jirovecii* and other infections in patients receiving immunosuppressive therapy [9, 18]. EULAR specifically recommended co-trimoxazole for this indication [9].

Rituximab impairs vaccine responses by depleting B cells, causing many patients to fail to show any antibody response to vaccination [75]. To maximize vaccination benefits, adjusting vaccination schedules is necessary. Apart from influenza vaccination, it is recommended to defer non-live attenuated vaccines until just before the next rituximab dose and delaying rituximab treatment for 2 weeks after vaccination. For coronavirus disease (COVID-19) vaccination, the recommended time window is 6 months after the last rituximab dose [76, 77].

## TIP 9. USE RITUXIMAB IN RELAPSING DISEASE

The EULAR consensus defines relapse as a recurrence of active disease after a period of remission [9]. A thorough review to exclude infection, malignancy, non-adherence to prescribed medications, or illicit drug abuse is needed when confirming relapse [78]. The occurrence of relapse is associated with development of ESKD and carries a poorer prognosis [79].

Rituximab is preferred as the immune suppressive of choice for relapsing disease, with dosing similar to initial induction regimens [9, 16, 18]. The RITAZAREM trial demonstrated a high rate of efficacy when using rituximab in relapsed patients [80].

Risk factors for relapse include PR3-ANCA positivity, ear nose and throat (ENT) disease, ANCA reappearance, detection of circulating B cells within a year after rituximab treatment, lower creatinine at baseline, and cardiovascular involvement [81, 82]. The PEXIVAS trial's *post hoc* analysis showed that plasma exchange or initial glucocorticoid regimen did not affect relapse risk [83].

## TIP 10. SWITCH WHEN DEALING WITH REFRACTORY DISEASE AND CONSIDER PLASMA EXCHANGE

Refractory disease has been defined as progressive disease despite standard therapy, failure to achieve remission by 6 months, or relapse on maintenance therapy [84]. As with a diagnosis of relapsing disease, it is important to reconsider the vasculitis diagnosis, search for vasculitis drives or triggers, and assess adherence and adequacy of therapy [9, 85].

Both KDIGO and EULAR guidelines recommend increasing the glucocorticoid dose and considering plasma exchange. While the KDIGO and American College of Rheumatology/Vasculitis Foundation (ACR/VF) guidelines recommended switching between rituximab or cyclophosphamide, the EULAR consensus does not comment on this approach. In selected patients, adding IV immunoglobulin can be an option. These cases should be managed at tertiary centres with expertise [9, 16, 18].

In our opinion, a biopsy should be considered to diagnose refractory disease and escalation of therapy is indicated. For patients at high risk of renal progression, we would consider plasma exchange as well as the combination of cyclophosphamide and rituximab treatment. The role of avacopan in refractory disease has not been established.

## Data Availability

No new data were generated or analysed in support of this work.
